# Theoretical Investigation of the NO_3_ Radical Addition to Double Bonds of Limonene

**DOI:** 10.3390/ijms10093743

**Published:** 2009-08-27

**Authors:** Lei Jiang, Wei Wang, Yi-Sheng Xu

**Affiliations:** 1 College of Water Sciences, Beijing Normal University, Beijing 100875, China; 2 Atmospheric Chemistry and Aerosol Research Division, Chinese Research Academy of Environmental Science, Beijing 100012, China; E-Mails:jiangle3657@sina.com (L.J.);xuys@craes.org.cn (Y.-S.X.)

**Keywords:** limonene, nitrate radical (NO_3_), ab initio, volatile organic compounds

## Abstract

The addition reactions of NO_3_ to limonene have been investigated using *ab initio* methods. Six different possibilities for NO_3_ addition to the double bonds, which correspond to the two C–C double bonds (endocyclic or exocyclic) have been considered. The negative activation energies for the addition of NO_3_ to limonene are calculated and the energies of NO_3_-limonene radical adducts are found to be 14.55 to 20.17 kcal mol-1 more stable than the separated NO_3_ and limonene at the CCSD(T)/6–31G(d) + CF level. The results also indicate that the endocyclic addition reaction is more energetically favorable than the exocyclic one.

## 1. Introduction

Total emissions of volatile organic compounds (VOCs) from vegetation have been estimated to be about 1,150 Tg carbon year^−1^ [1]. More than half (54%) of the emissions are related to isoprene and monoterpenes (~11%), such as α- and β-pinene and limonene [2]. Atmospheric oxidation of VOCs, which is initiated by reactions with a variety of oxidants including OH radical, NO_3_ radical and ozone is a common atmospheric chemistry reaction of the lower troposphere layer. During the day the major sinks for VOCs are their reactions with OH radical and O_3_, while with nitrate radical (NO_3_) can be a very important, and often dominant, loss process at nighttime [3–6]. The reactions of NO_3_ species, formed by the reaction of NO_2_ and O_3_ [7], with atmospheric VOCs can lead to formation of HNO_3_ [8], peroxyacyl nitrate (PAN) [9], RO_2_ [9,10], and toxic compounds such as dinitrates [10].

Limonene or 4-isopropenyl-1-methyl-cyclohexene is the most abundant monoterpene, and has both highly active endocyclic and exocyclic double bonds [11]. Plants non-native to Europe, *e.g.,* Australian eucalyptus, have limonene emissions of more than 30% of monoterpene [12] and limonene represents one of the four highest terpenes emitted in North America. Many reactions of monoterpenes with OH radical and O_3_ have received considerable attention with regard to their initiated oxidations, reaction kinetics, reaction products and secondary organic aerosol (SOA) formation whereas those with NO_3_ have received relatively little consideration, especially the reaction of limonene with NO_3_. The initial reaction of limonene and NO_3_ proceeds mainly by NO_3_ addition to the C–C double bond, forming the NO_3_-limonene radical adduct. Under atmospheric conditions, the NO_3_-limonene radical adduct is expected to react primarily with oxygen molecules to form the nitrooxyalkyl peroxy radicals, which further react with NO, engage in a self-reaction or crossreaction with other peroxy radicals, or react with HO_2_. The products and aerosol formation from the NO_3_ radical initiated oxidation of limonene have been investigated in the EUPHORE photoreactor facility [13]. In EUPHORE study, the results indicated that endolim had been identified as the major reaction products of the NO_3_ radical initiated oxidation of limonene and the total SOA mass formed occurs mainly through the secondary chemistry of its major product endolim. Hence, the nighttime reaction between limonene and NO_3_ contributes significantly to the degradation of limonene and SOA formed. However, such important processes and quantities as the formation of the NO_3_-limonene radical adduct and the isomer-specific reactions have yet to be clarified.

To examine the reaction mechanism of NO_3_ with limonene, a theoretical exploration of the NO_3_-initiated limonene oxidation mechanism with different possibilities for the initial step of the NO_3_ addition to the limonene was carried out and is reported here, as the isomeric branching of the initial NO_3_ addition to limonene is crucial in determining the final product distribution. The present study is mainly focused on comparison of different reaction pathways and determination energetically favorable bonds needed for the further elaboration of the oxidation mechanisms and assessment of the products identified in experiments. Moreover, the quantum chemical study can supply some insight on the reactions between NO_3_ and monoterpenes, especially important to understand the night-time atmospheric chemistry in the troposphere.

## 2. Theoretical Methods

The theoretical computations have been carried out on the SGI ALTIX 4700 supercomputer using the GAUSSIAN 03 suite of programs [14]. The geometry optimization of all reactants, transition states, and radical adducts was performed at UB3LYP/6–31G(d,p) with harmonic vibrational frequencies analyses. The stationary points were classified as minima in the case, when no imaginary frequencies were found, and as a transition state if only one imaginary frequency was obtained. The DFT optimized structures were then used in the single-point energy *ab initio* calculations using frozen core second-order Møller-Plesset perturbation theory (MP2) and coupled-cluster theory with single and double excitations including perturbative corrections for the triple excitations (CCSD(T) with various basis sets to obtain accurate energy information.

Density functional theory (DFT) has been widely employed for atmospheric oxidation reactions of VOCs with NO_3_ radical, OH radical and ozone [15–21]. These studies implied that deviations of the B3LYP geometries from the CASSCF ones are small enough to consider that the former level is a good compromise between quality and computational cost in the nitrate radical addition reaction and provides reasonable reaction energies and barriers for most of the ozonolysis steps insofar as the calculated B3LYP values are more accurate than those obtained with MP2 theory. So, to some degree, DFT was identified a reliable and economical method that provides a reasonable description of the VOCs-radical reactions.

In addition, for more accurate single-point energy, the basis set effects on calculated energies for NO_3_-limonene reactions were corrected at MP2 level according to the recently developed method by Zhang *et al.* in his investigation of the complex reaction mechanisms and pathways of various volatile organic compounds (VOCs) in the atmosphere [22]. A correction factor (CF) was determined from the energy difference between the MP2/6–31G(d) and MP2/6–311++G(d,p) levels. The values of calculated energies were then evaluated by CCSD(T)/6–31G(d) + CF method. The applied method has been validated for several isoprene reactions initiated by NO_3_, OH and O_3_ [16,22,23], and of limonene initiated by O_3_ [24].

## 3. Results and Discussion

**Figure 1.**
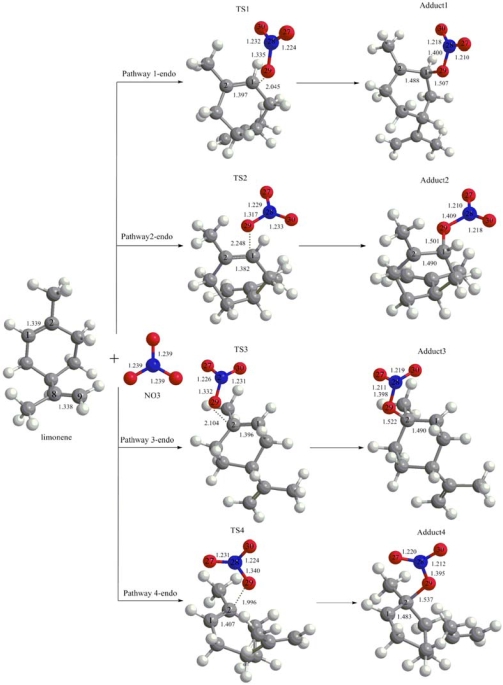
Geometries of stationary points involved in the NO_3_ addition to limonene endocyclic double bond obtained at UB3LYP/6-31G(d,p) level of theory. Bond distances are given in Å. TS1, TS2, TS3, and TS4 are abbreviations for 1-endo, 2-endo, 3-endo and 4-endo transition states, respectively.

**Figure 2.**
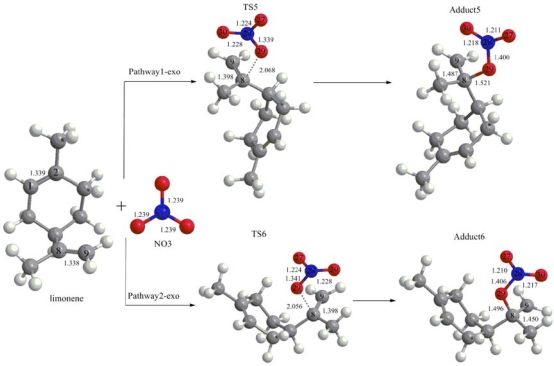
Geometries of stationary points involved in the NO_3_ addition to limonene exocyclic double bond obtained at UB3LYP/6–31G(d,p) level of theory. Bond distances are given in Å. TS5 and TS6 are abbreviations for 1-exo and 2-exo transition states, respectively.

The computational results show that there are six possibilities for the NO_3_ upon addition to the double bond of the limonene, namely, four possibilities involving the endocyclic C–C double bond (see Figure 1) and two possibilities for the exocyclic C–C double bond (see Figure 2). Transition states are not found in the evaluated pathways of the NO_3_ addition to limonene, which proceed in the direction of the terminal carbon atom (C9) of the exocyclic C–C double bond. This can be explained by the stronger selectivity of the NO_3_ and the initial step proceeds predominantly via electrophilic addition mechanism for the reactions of NO_3_ with alkenes [13,25,26].

### 3.1. Reaction Mechanism

*T*_1_ diagnostic values, spin eigenvalues of the unrestricted wavefunction (<S^2^>) and its projection (<S^2^>A) for all stationary points in the NO_3_ addition to the limonene are provided in Table 1. All calculations of *T*_1_ diagnostic values were run at CCSD(T)/6–31G(d)//UB3LYP/6–31G(d,p) level of theory. *T*_1_ diagnostic values give a qualitative assessment of the nondynamical correlation significance and ensure the reliability of this treatment. For closed-shell systems, *T*_1_ values should be under 0.02 and for open-shell systems under 0.045 [27,28].

As seen from Table 1, the closed-shell *T*_1_ diagnostic value is 0.0100 for limonene, and the the largest open-shell *T*_1_ diagnostic value is 0.0289 for TS2. Hence, *T*_1_ values calculated for all the stationary points are clearly under these thresholds. At the UB3LYP/6–31G(d,p) level of theory, the calculated spin eigenvalues, <S^2^>, are 0.754 and 0.768 for radicals NO_3_, TS1–TS6 and Adduct1–Adduct6, respectively. After proper projection, the spin eigenvalues are reduced to 0.750 for all the open-shell stationary points, indicating that contamination of the unrestricted Hartree-Fock wave function from higher spin states is negligible.

Figure 1 presents the optimized geometries of the stationary points for the NO_3_ addition to the endocyclic C–C double bond obtained at the UB3LYP/6–31G(d,p) level of theory and values of the most important geometrical properties.

As seen from this Figure, the NO_3_ addition reaction can proceed either on the H-substituted carbon atom (C1), or on the methyl-substituted carbon atom (C2). On each carbon atom of relevant double bond, there are two possible attacking directions. Four transition states (TS1, TS2, TS3 and TS4) associated with the addition of NO_3_ to the endocyclic C–C double bond leading to the formation of the NO_3_-limonene radical adducts (Adduct1–Adduct4) have been identified. Each transition state has only one imaginary harmonic vibrational frequency and can be classified as the first-order saddle point. The values of imaginary frequencies for TS1, TS2, TS3, and TS4 transition states are 157.10i, 67.07i, 157.82i, and 228.95i, respectively.

In the 1-endo pathway, the C–C double bond distance (1.339 Å), increases by 0.058 Å and 0.149 Å in the transition states (TS1) and NO_3_-limonene radical adduct (Adduct1), respectively, which clearly demonstrates that a single bond property on the formation of the NO_3_-limonene radical adducts presented during the reaction procedure. The disappearance of the double bond is also shown in the reaction channel. The NO_3_-limonene distance in the TS1 is 2.045 Å, while in the Adduct1 is 1.507 Å. In NO_3_ radical, the three N–O bond lengths are the same values (1.239 Å). While in the TS1, the length of the N–O bond oriented towards the C–C double bond, increases a length of 0.096 Å and becomes to 1.400 Å for the Adduct1. Meanwhile it shows decreasing tendency to the other two bonds. Compared with those in the NO_3_ radical, the bond lengths have less variation and decrease 0.007–0.029 Å in the TS1 and Adduct1. Other three pathways of transition states (TS2, TS3 and TS4) and NO_3_-limonene radical adducts (Adduct2, Adduct 3 and Adduct 4) exhibit a similar pattern.

The optimized geometries for the NO_3_ addition to the exocyclic C–C double bond are illustrated in Figure 2. In this case, the NO_3_ addition could proceed on the methyl-substituted carbon atom (C8). Two transition states (TS5 and TS6) associated with the addition of NO_3_ to the exocyclic C–C double bond leading to the formation of the NO_3_-limonene radical adducts (Adduct5 and Adduct6) have been identified too. The values of imaginary frequencies for TS5 and TS6 transition states are 191.62i and 231.50i, respectively.

In comparison of the 1-endo pathway, the 1-exo and 2-exo pathways exhibited a similar addition mode are shown in Figure 2. The C–C double bond distance (1.338 Å), increases by a length of 0.06 Å in the transition states (TS), and to 1.450–1.487 Å in the NO_3_-limonene radical adducts, and clearly becomes a single bond. The NO_3_-limonene radical distance in the two TS is in the range of 2.056–2.068 Å, and in the two adduct isomers is between 1.496 and 1.521 Å. In the TS, the length of the N–O bond oriented towards the C–C double bond, increases lengths in the range of 0.1–0.102 Å and increases to 1.400–1.406 Å in the adduct isomers. While the other two N–O bonds lengths vary less and decrease 0.011–0.029 Å in the TS and adduct isomers.

### 3.2. Thermochemical Analysis

The NO_3_-limonene reaction energies computed at different levels of theory with different basis sets with the zero-point correction included are presented in Table 2. As seen from this data, the reaction energies of the NO_3_ addition to limonene calculated at different levels of theory are quite different. The values predicted by MP2/6–31G(d) and B3LYP/6–311 + G(3df,2pd) are comparable and differ by −2.7–2.28 kcal mol^−1^. The values obtained at CCSD(T)/6–31G(d) level theory are 5.32–13.52 kcal mol^−1^ more stable than those calculated with MP2 and B3LYP. Another important detail is that the values determined by using MP2 and B3LYP and with two different basis sets differ by 1.71–2.73 kcal mol^−1^ and 3.22–4.06 kcal mol^−1^, respectively. At the CCSD(T)/6–31G(d) + CF level of theory, the NO_3_-limonene radical adducts are 14.55–20.17 kcal mol^−1^ more stable than the separate NO_3_ and limonene. Adduct2 is more stable than others, The difference in the relative stability of the six NO_3_-limonene radical adducts does not exceed 5.62 kcal mol^−1^.

The NO_3_-limonene activation energies with the zero-point correction (ZPE) included computed at different levels of theory with different basis sets, are given in Table 3. As seen from this Table, the activation energies for the formation of the NO_3_-limonene reactions with the zero-point correction included are very sensitive to the effects of electron correlation and basis set. The activation energies of the NO_3_-limonene reaction are positive activation energies and significantly overestimate with MP2 method. B3LYP/6–311 + G(d,p), B3LYP/6–311 + G(3df,2pd) (except for *ΔE_2-exo_*), CCSD(T)/6–31G(d) and CCSD(T)/6–31G(d) + CF (except for *ΔE_2-exo_*) levels are negative activation energies, which corresponds to the NO_3_ + propene reaction, ozonolysis of isoprene and the OH + ethane reaction reported in the previous theoretical studies [15,22,29,30]. The negative activation energies given by B3LYP/6–311 + G(3df,2pd) and CCSD(T)/6–31G(d) are comparable, with a largest difference of 4.04 kcal mol^−1^ for *ΔE_2-exo_*. Compared with CCSD(T)/6–31G(d) + CF, there are noticeable differences in the calculated activation energies for the NO_3_-limonene reaction with the UB3LYP/6–31G(d,p) methods of and results differ by 1.12–5.98 kcal mol^−1^, while there are little differences with B3LYP/6–311 + G(3df,2pd), and they differ by −0.6–3.32 kcal mol^−1^. The activation energies obtained at the CCSD(T)/6–31G(d) + CF level of theory are −3.47 kcal mol^−1^ for 1-endo, −2.03 kcal mol^−1^ for 2-endo, −4.36 kcal mol^−1^ for 3-endo, −3.60 kcal mol^−1^ for 4-endo, −1.43 kcal mol^−1^ for 1-exo and 1.67 kcal mol^−1^ for 2-exo. This indicates that 3-endo is the most favorable pathway. It also implies that the endocyclic addition is more energetically favorable than the exocyclic one and that the NO_3_ attack predominantly takes place at the endo-double bonded carbons.

**Figure 3.**
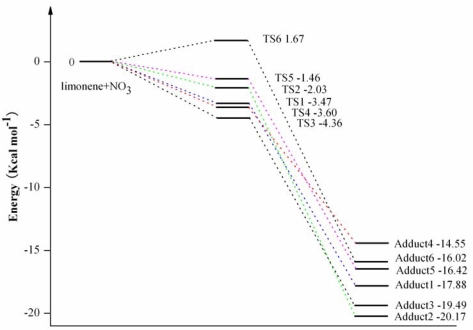
NO_3_-limonene reaction coordinates: relative energies of the stationary points located on the separate NO_3_ and limonene ground-state potential energy surface. The energy values are given in kcal mol^−1^ and are calculated using CCSD(T)/6–31G(d) + CF//UB3LYP/6–31G(d,p).

As seen from Table 3, relatively low activation or slightly negative energies obtained for the reaction of NO_3_ with limonene in our work, which agree with the values obtained for the reaction of NO_3_ with alkenes and other terpenes. Hence, the reaction of the NO_3_ addition to limonene is entirely consistent with a radical addition process [25]. Figure 3 illustrates the relative energies of the stationary points located on the singlet ground-state separate NO_3_ and limonene potential energy surface at the CCSD(T)/6–31G(d) + CF level of theory.

The NO_3_-limonene reaction enthalpies, Gibbs free energies and entropies computed at B3LYP/6–31G(d,p) level of theory with the thermal Correction included, are shown in Table 4. The NO_3_-limonene addition reaction is exothermic for the six pathways. The six radical adducts (Adduct1–Adduct6) are 9.32–17.42 kcal mol^−1^ more stable than the reactants. The endocyclic addition reactions are more stabilized than the exocyclic addition reaction, except for the 4-endo pathway. In the endocyclic addition reactions, the Adduct2, corresponding to the 2-endo pathway, is the most stabilized, with value of −17.42 kcal mol^−1^. Adduct5 is more stabilized, with a value of −11.73 kcal mol^−1^, corresponding to the 1-exo pathway in the exocyclic addition reactions. The NO_3_-limonene reaction Gibbs free energies range from −4.69 to 3.61 kcal mol^−1^ for the six pathways. The values of Gibbs free energies are negative for 1-endo, 2-endo and 3-endo pathways, implying that the reaction of NO_3_ with limonene can take place spontaneously.

Additionally, the formation of a prereactive van der Waals complex has been used to explain the negative values found for the experimental activation energies [31]. A van der Waals complex was formed prior to addition have been reported in quantum chemical studies on the OH addition reaction to alkenes [30,32,33]. A transition state connecting the van der Waals complex with the adduct has also been found. But, as for the case of the propene + NO_3_ reaction, no van der Waals complexes in the limonene + NO_3_ reaction have been found at the UB3LYP/6–31G(d,p) level of calculation, since the DFT method fails in case of Van der Waals and loose transition states and actually represent only small irregularities on the potential energy hypersurface [15]. The van der Waals complexes do not have a profound effect on the kinetics [30] and are not chemically relevant in the reaction mechanisms [34], although they will probably exist.

## 4. Conclusions

Our theoretical investigation reveals several important aspects regarding the gas phase reaction of the initial NO_3_ addition to the limonene using computational quantum, which has importance in nighttime atmospheric chemistry.

(1) The activation energies for the formation of the NO_3_-limonene reaction have been determined first. At CCSD(T)/6–31G(d) + CF//UB3LYP/6–311 + G(d,p), our results indicate that relatively low activation or slightly negative energies were obtained, and 3-endo pathway is the most favorable branching for the initial step of the NO_3_-limonene reactions. From the obtained activation energies, we concluded that the reaction of the NO_3_ addition to limonene is entirely consistent with a radical addition process.

(2) The calculated activation energies of the NO_3_-limonene reactions are very sensitive to electron correlation and basis set effects. The activation energies of the NO_3_-limonene reaction were significantly overestimated with the MP2 method. Compared with CCSD(T)/6–31G(d) + CF, there are noticeable differences in the calculated activation energies for the NO_3_-limonene reaction with the methods of UB3LYP/6–31G(d,p), while there was little difference with B3LYP/6–311 + G(3df,2pd).

(3) Six possibilities for the NO_3_ addition to double bond on the limonene were found; the two possibilities for the NO_3_ addition proceeding on the terminal carbon atom (C9) of the exocyclic C–C double bond are not found. The endocyclic addition is more energetically favorable than the exocyclic one. Those are consistent with the EUPHORE study by Spittler *et al.,* who could not detect the products formaldehyde or 4-acetyl-1-methylcyclohex-1-ene, an indication of an attack of NO_3_ on the exocyclic double bond in limonene. Hence, the computation results verified that the high selectivity of NO_3_ towards different C–C double bonds and the initial step proceeds predominantly via electrophilic addition for the reactions of NO_3_ with limonene.

## Figures and Tables

**Table 1.** *T*_1_ diagnostic values, spin eigenvalues of the unrestricted wavefunction and its projection for all stationary points in the NO_3_ addition to the limonene.^a^

**Species**	**CCSD(T)/6–31G(d)**	**UB3LYP/6–31G(d,p)**	**UB3LYP/6–31G(d,p)**

	***T*_1_**	**<S^2^>**	**<S^2^>A**
limonene	0.0100	0.000	0.000
NO_3_	0.0228	0.755	0.750
TS1	0.0211	0.761	0.750
Adduct 1	0.0148	0.754	0.750
TS2	0.0289	0.760	0.750
Adduct 2	0.0149	0.754	0.750
TS3	0.0230	0.764	0.750
Adduct 3	0.0149	0.754	0.750
TS4	0.0202	0.764	0.750
Adduct 4	0.0156	0.754	0.750
TS5	0.0220	0.768	0.750
Adduct 5	0.0146	0.754	0.750
TS6	0.0215	0.768	0.750
Adduct 6	0.0145	0.754	0.750

^a^ All optimized geometries calculated at the UB3LYP/6–31G(d,p) level.

**Table 2.** NO_3_-limonene Reaction Energies (RE) with Zero-Point Correction Included (kcal mol^−1^) computed at different levels of theory for the six pathways a.

**Method**	***RE_1-endo_***	***RE_2-endo_***	***RE_3-endo_***	***RE_4-endo_***	***RE_1-exo_***	***RE_2-exo_***
PMP2/6–31G(d)	−9.76	−12.19	−11.41	−5.68	−9.15	−8.72
PMP2/6–311++G(d,p)	−7.06	−9.46	−8.99	−3.97	−6.45	−6.18
B3LYP/6–31G(d,p)	−15.26	−16.90	−14.00	−8.73	−11.26	−10.08
B3LYP/6–311 + G(3df,2pd)	−12.04	−13.15	−10.43	−4.91	−7.25	−6.02
CCSD(T)/6–31G(d)	−20.58	−22.90	−21.90	−16.26	−19.12	−18.55
CCSD(T)/6–31G(d) + CF	−17.88	−20.17	−19.49	−14.55	−16.42	−16.02

^a^ optimized geometries, vibrational frequencies and ZPE obtained at the UB3LYP/6–31G(d,p) level.

**Table 3.** NO_3_-limonene Activation Energies (ΔE) with Zero-Point Correction Included (kcal mol^−1^) computed at different levels of theory for the six pathways a.

**Method**	***ΔE_1-endo_***	***ΔE_2-endo_***	***ΔE_3-endo_***	***ΔE_4-endo_***	***ΔE_1-exo_***	***ΔE_2-exo_***
PMP2/6–31G(d)	7.20	13.68	7.68	7.15	8.62	11.06
PMP2/6–311++G(d,p)	10.06	16.06	10.55	9.41	11.94	14.50
B3LYP/6–31G(d,p)	−8.17	−8.01	−7.72	−4.72	−4.34	−0.92
B3LYP/6–311 + G(3df,2pd)	−6.47	−5.35	−5.46	−2.02	−1.30	2.27
CCSD(T)/6–31G(d)	−6.34	−4.41	−7.23	−5.86	−4.78	−1.77
CCSD(T)/6–31G(d) + CF	−3.47	−2.03	−4.36	−3.60	−1.46	1.67

^a^ optimized geometries, vibrational frequencies and ZPE obtained at the UB3LYP/6–31G(d,p) level.

**Table 4.** NO_3_-limonene Reaction Enthalpies, Gibbs Free Energies and Entropies (ΔH and ΔG in kcal mol^−1^, ΔS in cal mole^−1^K^−1^) with Thermal Correction Included computed at B3LYP/6–31G(d,p) level of theory for the six pathways a.

**Method**	**1-endo**	**2-endo**	**3-endo**	**4-endo**	**1-exo**	**1-exo**
*ΔH*	−15.59	−17.42	−14.70	−9.32	−11.73	−10.55
*ΔG*	−3.64	−4.69	−0.97	3.61	1.80	2.83
*ΔS*	−40.08	−42.70	−46.05	−43.37	−45.38	−44.88

^a^ optimized geometries, vibrational frequencies and Thermal Correction to Enthalpy and Gibbs Free Energy obtained at the UB3LYP/6–31G(d,p) level.
